# Comparison of survival patterns of northern and southern genotypes of the North American tick *Ixodes scapularis* (Acari: Ixodidae) under northern and southern conditions

**DOI:** 10.1186/1756-3305-7-394

**Published:** 2014-08-26

**Authors:** Howard S Ginsberg, Eric L Rulison, Alexandra Azevedo, Genevieve C Pang, Isis M Kuczaj, Jean I Tsao, Roger A LeBrun

**Affiliations:** USGS Patuxent Wildlife Research Center, RI Field Station, Woodward Hall – PSE, University of Rhode Island, Kingston, RI 02881 USA; Department of Plant Sciences and Entomology, Woodward Hall, University of Rhode Island, Kingston, RI 02881 USA; Caesar Kleberg Wildlife Research Institute, Texas A&M University-Kingsville, Kingsville, TX 78363 USA; Department of Fisheries and Wildlife, Michigan State University, East Lansing, MI 48824 USA

**Keywords:** *Ixodes scapularis*, Survival, Northern genotypes, Southern genotypes, Geographical gradients

## Abstract

**Background:**

Several investigators have reported genetic differences between northern and southern populations of *Ixodes scapularis* in North America, as well as differences in patterns of disease transmission. Ecological and behavioral correlates of these genetic differences, which might have implications for disease transmission, have not been reported. We compared survival of northern with that of southern genotypes under both northern and southern environmental conditions in laboratory trials.

**Methods:**

Subadult *I. scapularis* from laboratory colonies that originated from adults collected from deer from several sites in the northeastern, north central, and southern U.S. were exposed to controlled conditions in environmental chambers. Northern and southern genotypes were exposed to light:dark and temperature conditions of northern and southern sites with controlled relative humidities, and mortality through time was recorded.

**Results:**

Ticks from different geographical locations differed in survival patterns, with larvae from Wisconsin surviving longer than larvae from Massachusetts, South Carolina or Georgia, when held under the same conditions. In another experiment, larvae from Florida survived longer than larvae from Michigan. Therefore, survival patterns of regional genotypes did not follow a simple north–south gradient. The most consistent result was that larvae from all locations generally survived longer under northern conditions than under southern conditions.

**Conclusions:**

Our results suggest that conditions in southern North America are less hospitable than in the north to populations of *I. scapularis*. Southern conditions might have resulted in ecological or behavioral adaptations that contribute to the relative rarity of *I. scapularis* borne diseases, such as Lyme borreliosis, in the southern compared to the northern United States.

## Background

The blacklegged tick, *Ixodes scapularis* Say (Acari: Ixodidae), is the primary North American vector of *Borrelia burgdorferi* Johnson, Schmid, Hyde, Steigerwalt & Brenner, etiologic agent of Lyme disease, which is the most commonly-reported vector-borne disease in the United States [1]. This tick is abundant in much of the northeastern, mid-Atlantic and north central United States, and is broadly distributed among the southeastern and south central states [2, 3]. In this paper, “north” refers to the areas of eastern and central North America north of the mouth of the Chesapeake Bay (37° N latitude), while “south” refers to areas south of that latitude.

Population genetics studies in the past two decades have revealed a complex geographical pattern. One widespread *I. scapularis* lineage, the American Clade, predominates in the north and is also broadly distributed in southern states [4]. In the southern states however, there is greater genetic diversity, including several Southern clades that are rarely found in the north [5, 6]. This pattern could have resulted from a founder effect during recent range expansion of *I. scapularis* following northward glacial recession [7, 8], or perhaps more recently, following broadscale landscape changes such as forest regrowth and expanding deer populations [9]. To date, the ecological characteristics of these various genetic groups have not been described.

Temperature and relative humidity (RH) are fundamental environmental factors that influence tick survival [10–12]. Laboratory studies have confirmed the relationship between RH and survival of *I. scapularis*, and have estimated the critical equilibrium activity RH for nymphs [13, 14]. Critical equilibrium activity RH is the level below which ticks lose water, thus possibly affecting survival [15]. Field studies indicate that survival in *I. scapularis* is greater in forested than in open habitats [16] and shows clear associations with temperature and humidity variables [17]. Furthermore, temperature and RH were related to summer activity in nymphal *I. scapularis* at a forest site in New Jersey [18], and influenced aspects of nymphal questing behavior in lab trials [19]. In view of the distributional pattern of genetic lineages of *I. scapularis* and the broad environmental differences between northern and southern regions, these results raise the question of whether the different genetic groups respond differently to the environmental conditions found in different regions of the eastern and central U.S.

In this study, we compare immature *I. scapularis* from northern genotypes (derived from adults collected at northeastern and north central locations) to ticks of southern genotypes (from multiple southern sites) in terms of survival under both northern and southern conditions of diurnal cycle and temperature, and at varying levels of relative humidity. We test three hypotheses about survival patterns of these ticks. The null hypothesis (***H***_***0***_) is that there is no difference in survival patterns between northern and southern ticks. The first alternative hypothesis (***H***_***1***_) is that the genetic groups are regionally adapted such that northern ticks survive longer than southern ticks under northern conditions, and southern ticks survive longer than northern ticks under southern conditions. The second alternative hypothesis (*H*_*2*_) is that the northern ticks, which come from the widely distributed northern genotypes, are hardier than the southern genotypes and survive longer than southern ticks under both northern and southern conditions. We assess variability of survival among different genotypes within as well as between different regions.

## Methods

We tested the survival patterns of northern compared to southern genotypes of *I. scapularis* in three series of experiments (details below). The pilot study, experiment 1, compared survival of both larvae and nymphs of *I. scapularis* from one northern site and one southern site, under both northern and southern temperature and daylength conditions, at both high and low relative humidities. Experiment two assessed variability within and between regions by testing survival of larvae from three different mothers from each site, and by testing ticks from additional northern and southern locations. We held larvae at high humidity for experiment 2 because that group showed a good spread in experiment 1. Experiment 3 tested larvae from three different mothers from each of two additional locations. We utilized an intermediate humidity level to determine whether the patterns observed in experiment 2 would differ at different humidities.

### Experiment 1

*Ixodes scapularis* larvae were obtained from laboratory colonies maintained at Michigan State University. The northern colony originated from engorged adult ticks collected from white tailed deer in Wisconsin (hereafter, WI_*1*_). The southern colony originated from engorged adults collected from deer in South Carolina (SC). Offspring of those adults were reared (on mouse hosts) to adults that were fed on rabbits. All handling of animals in this study was performed in accordance with IACUC approval from Michigan State University (# 06/12-103-00). Larvae that were the offspring from one adult of each clade were used in this study. Egg hatch for the northern ticks started on 7 February 2012, and egg hatch for the southern ticks started on 5 February 2012. The nymphs were derived from mouse-fed larvae, and individuals from both clades (originally from one southern female and four northern females) emerged from 19 April through 18 May 2012. The genetic characteristics of the colonies were determined at Georgia Southern University, using the clade classification developed by L. Beati, under which our northern ticks belonged to American Clade 1, and our southern ticks were Southern Clade 1 [6].

For the larval experiment, northern and southern conditions were based on light:dark and temperature cycles during the dates of peak larval activity in the north and south (based on 2011 field data). One Percival I-36LL environmental unit (Percival Scientific, Perry, IA) was set at northern conditions, based on average conditions at the Chatham weather station on Cape Cod, MA on 1 August (similar to those in Racine, WI), and a second environmental unit was set at southern conditions based on an Aiken, SC weather station on 15 June. Northern conditions were L:D 14.5:9.5 hrs, day 23.3°C: night 16.6°C. Southern conditions were L:D 14.4:9.6 hrs, day 32.3°C, night 18.3°C. The chambers switched abruptly between day and night conditions. For the nymphal experiment, northern settings were based on Chatham conditions on 15 June (15.25:8.75 L:D, 19.4:12.2°C), and southern settings were based on Aiken conditions on 15 July (14.17:9.83 L:D, 34.4:20.5°C).

Within each environmental unit, two humidity chambers were maintained at high humidity (using deionized water) and two chambers at low humidity (using saturated NaCl solutions), to produce RH of about 90% and 75% in the two treatments [20]. These RH values were selected because the critical equilibrium activity RH for *I. scapularis* nymphs, below which survival is affected, is about 82-85% [13, 14]. Eight to ten plastic jars (3-dram snap-cap vials; IntraPac, Plattsburgh, NY) with screen tops were placed in each humid chamber, with an average of about 5 ticks per jar. This approach allowed observers to easily detect mortality of individual ticks in each jar, to count totals for each treatment, and to provide estimates of variability in mortality among jars. Ticks of the two clades were randomly assigned to treatments, such that ticks from each clade were placed in high and low RH chambers under northern and southern conditions. The larval trial was run on 21–26 May, and the nymphal trial on 11–17 June 2012. Survival was determined by tapping each jar on the tabletop, breathing through the screen top, and then observing under a dissecting microscope for evidence of any movement. The numbers of live and dead ticks were recorded. Observations were made every eight hours for 48 hours after initial setup, and every 24 hours thereafter. Temperature and humidity were recorded before each reading using Fisher Scientific Traceable Humidity/Temperature Pens (Control Company, Friendswood, TX) that were maintained within each chamber.

Survival through time was analyzed by comparing cumulative mortality between treatments using Kolmogorov-Smirnov two-sample tests [21], which assess whether the point of greatest difference between two cumulative distributions is large enough to consider the distributions different. Two-tailed tests were used to test the null hypothesis (no difference between treatments), and one-tailed tests were used to test the alternative hypotheses (survival was greater in one treatment than another).

### Experiment 2

**Figure 1:**
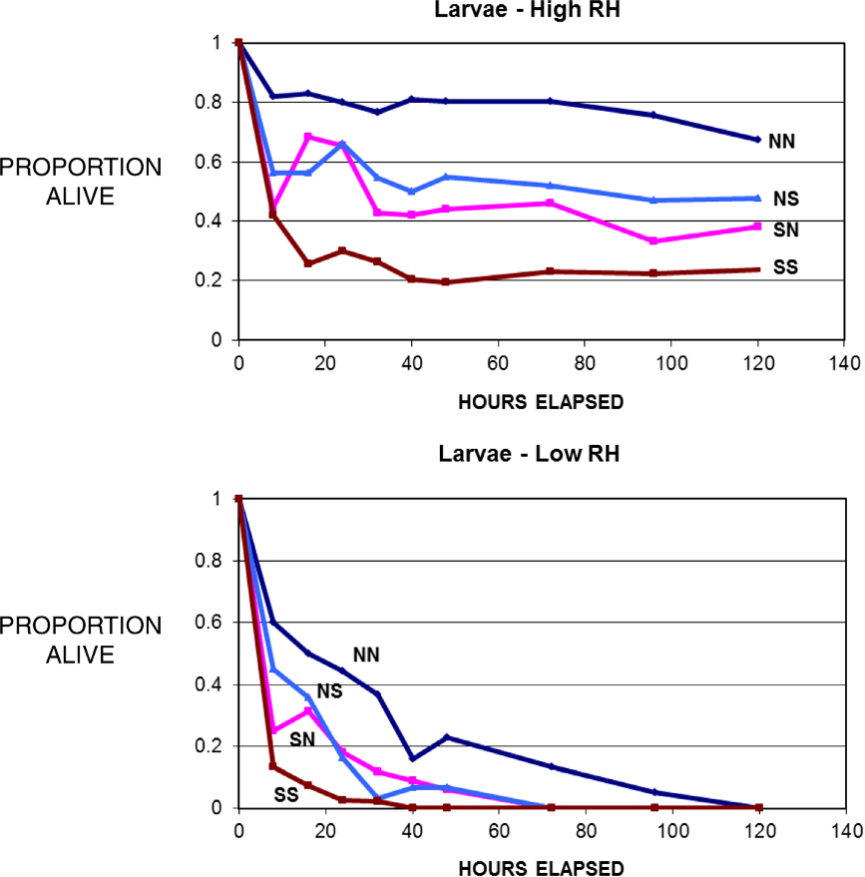
**Survival of** ***Ixodes scapularis*** **larvae under conditions of high and low relative humidity.** NN = northern (WI) ticks under northern conditions; NS = northern ticks under southern conditions; SN = southern (SC) ticks under northern conditions; SS = southern ticks under southern conditions.

We studied variability in survival of immature *I. scapularis* within and between geographical regions by testing larvae from three different mothers from each of three different locales. We used larvae at high humidity because this level provided a broad spread in the results of Experiment 1 (Figure 1). The larvae hatched in the lab at Michigan State University from eggs laid by engorged females that had been collected from white tailed deer at check stations in Fort McCoy, Wisconsin (WI_*2*_), Middleboro, Massachusetts (MA), and Bulloch County, Georgia (GA).

Egg hatch started from 20–28 December 2012 for the GA ticks, but from 29 January – 11 February 2013 for the WI_*2*_ and MA ticks, so to insure that the larvae were roughly the same age, we started the survival trials for the GA larvae on 8 April, and for the WI_*2*_ and MA larvae on 23 May 2013. Larvae were held at high humidity, 20-24°C and ambient light/dark before the survival trials. We placed a clump of approximately 10 larvae in each screen-top jar, and randomly placed jars into humid chambers and treatments (three chambers each in northern and southern treatments) such that there were three jars with larvae from each mother in each chamber. Northern and southern conditions were as in Experiment 1. Jars were examined for tick survival every 12 hours for the first 48 hours, daily for the next 6 days, every third day for the following 2 readings, and weekly for the following 2 weeks, for a total of 28 days (672 hours).

Survival was compared by analyzing the proportion alive in each jar at the end of the experiment using ANOVA with arcsine transformed proportions (SAS, version 9.3, GLM subprogram). The model included terms for northern vs. southern conditions, region (=state) of origin, clutch (larvae from each of the three females) nested within region, and interaction of N/S conditions with region of origin.

### Experiment 3

We assessed the generality of results from the previous experiments by testing survival of larvae from two additional locales and holding them at intermediate RH levels (attained using saturated KCl solutions in the humid chambers). The northern larvae came from three laboratory rabbit-fed females from Michigan (MI), and the southern larvae were from three laboratory rabbit-fed females from northern Florida (FL). FL egg hatch was from 1–17 May, while MI egg hatch was from 21 June – 4 July, so to insure that the larvae were roughly the same age, we started the FL survival trial on 19 July and the MI survival trial on 12 September 2013. Larvae were placed in screen-top jars within humid chambers as in Experiment 2, and jars were examined for tick survival according to the same schedule. Data were analyzed as in Experiment 2.

## Results

### Experiment 1

*Larvae.* Daytime humidity averaged about 91% in the high RH chambers and 74% in the low RH chambers (Table 1). At least some larvae survived in all treatments under high RH conditions, but not under low RH (Figure 1). Survival differed significantly between WI_*1*_ and SC larvae under conditions of both high RH (Kolmogorov-Smirnov Test, 2-tailed, *n*_*A1*_ = 94, *n*_*S1*_ = 88, ***D*** = 0.337, ***p*** < 0.001) and low RH (*n*_*A1*_ = 68, *n*_*S1*_ = 72, ***D*** = 0.343, p < 0.001). Therefore, the null hypothesis is rejected. ***H***_***1***_ is also rejected, because survival of SC larvae was not greater than that of WI_*1*_ larvae under southern conditions (Figure 1). In fact, WI_*1*_ survival was consistently higher than that of SC larvae under both high RH (Kolmogorov-Smirnov 1-tailed test, ***χ***^***2***^ = 20.621, ***p*** < 0.001) and low RH (***χ***^***2***^ = 16.443, ***p*** < 0.001) conditions (Figure 1). These results support ***H***_***2***_, that the broader ranging WI_*1*_ (American Clade) larvae are generally hardier than SC (Southern Clade) larvae. The apparent upward “blips” in some of the survival curves presumably resulted from ticks that were immobile during a reading, but not dead, and were active during a subsequent reading, or perhaps from occasional counting errors.

**Table 1:** **Daytime physical conditions in treatment chambers; experiment 1**

	Northern conditions						Southern conditions					
	High RH			Low RH			High RH			Low RH		
	Mean	SE	N	Mean	SE	N	Mean	SE	N	Mean	SE	N
Larvae												
Temp (°C)	22.87	0.130	14	22.41	0.051	14	32.80	0.118	14	34.39	0.396	14
RH (%)	90.20	0.442	10	75.14	0.417	14	91.57	2.021	14	72.57	0.768	14
Nymphs												
Temp (°C)	18.85	0.085	16	18.76	0.084	16	34.83	0.112	16	34.81	0.073	16
RH (%)	93.56	0.302	16	71.94	0.924	16	91.88	0.935	16	72.93	0.497	14

WI_1_ larvae had higher survival under northern than southern conditions at both high RH (Kolmogorov-Smirnov test, 1-tailed, *n*_*N*_ = 46, *n*_*S*_ = 48, ***D*** = 0.309, ***χ***^***2***^ = 8.94, df = 2, ***p*** < 0.025) and low RH (*n*_*N*_ = 38, *n*_*S*_ = 30, ***D*** = 0.333, ***χ***^***2***^ = 7.478, ***p*** < 0.025). Similarly, SC larvae had higher survival under northern than southern conditions at high RH (*n*_*N*_ = 51, *n*_*S*_ = 37, ***D*** = 0.430, ***χ***^***2***^ = 15.849, ***p*** < 0.001). The direction of the difference was the same at low RH (Figure 1), but was not significant (*n*_*N*_ = 33, *n*_*S*_ = 39, ***D*** = 0.238, ***χ***^***2***^ = 4.033, ***p*** > 0.1). Thus, survival was generally higher for both clades under northern than under southern conditions.

**Figure 2:**
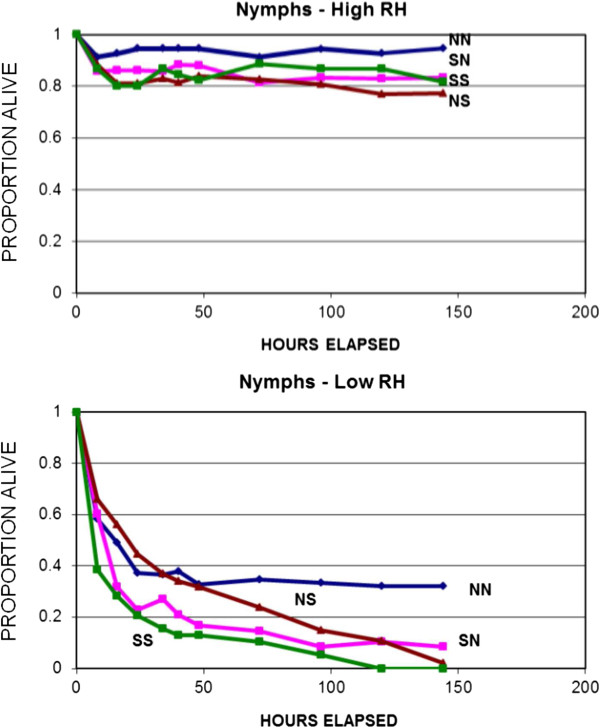
**Survival of** ***I. scapularis*** **nymphs under conditions of high and low relative humidity.** Labels as in Figure 1.

*Nymphs.* Daytime humidity averaged about 93% in the high RH chambers and 72% in the low RH chambers (Table 1). Survival did not differ significantly between WI_*1*_ and SC nymphs under conditions of high RH (Kolmogorov-Smirnov Test, 2-tailed, *n*_*A1*_ = 109, *n*_*S1*_ = 87, ***D*** = 0.051, ***p*** > 0.1). However, overall survival of the two clades differed under conditions of low RH (*n*_*A1*_ = 97, *n*_*S1*_ = 87, ***D*** = 0.223, p < 0.05) (Figure 2). Therefore, nymphal survival was high for both clades under both northern and southern conditions when RH was high, and clades differed in survival only when RH was low. ***H***_***1***_ is rejected, because survival of SC nymphs was not greater than that of WI_*1*_ under southern conditions (Figure 2). In fact, WI_*1*_ nymphal survival was significantly greater overall than that of SC under low RH conditions (Kolmogorov-Smirnov test, one-tailed, ***χ***^***2***^ = 9.117, ***p*** < 0.005). Therefore nymphal survival did not differ consistently between clades at high RH, but was similar to larval survival under low RH, with WI_*1*_ showing significantly higher survival than SC nymphs.

Survival at low RH was not significantly greater under northern than under southern conditions for either the WI_*1*_ (Kolmogorov-Smirnov test, 1-tailed, *n*_*N*_ = 51, *n*_*S*_ = 46, ***D*** = 0.214, ***χ***^***2***^ = 4.415, df = 2, ***p*** > 0.1) or the SC nymphs (*n*_*N*_ = 48, *n*_*S*_ = 39, ***D*** = 0.220, ***χ***^***2***^ = 4.149, ***p*** > 0.1). For both groups of nymphs, survival was similar under northern and southern conditions.

### Experiment 2

**Figure 3:**
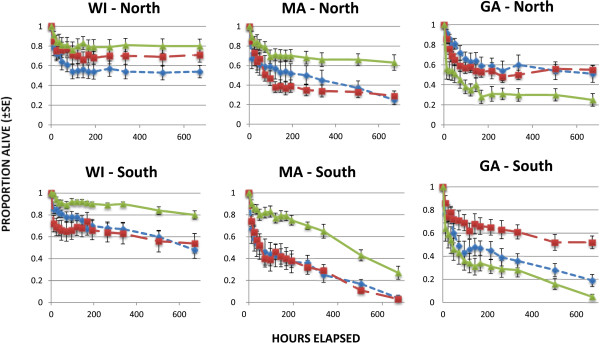
**Survival of** ***I. scapularis*** **larvae from three different mothers from each of three different regions under northern and southern conditions at high relative humidity.** Error bars are ± SE.

**Figure 4:**
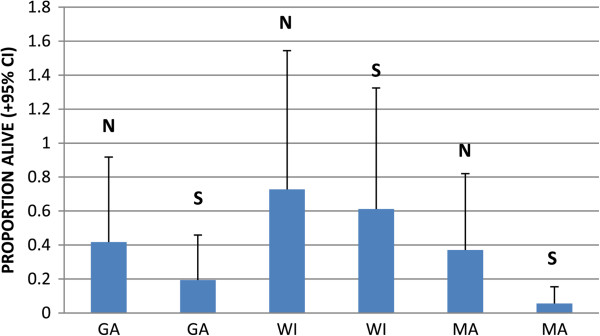
**Mean proportion alive after 28 days (+95% CI) of larvae from Georgia (GA), Wisconsin (WI), and Massachusetts (MA) under northern (N) and southern (S) conditions.** Means backtransformed from arcsine transformed data.

Daytime temperatures were in the range of 22-23°C in the chambers simulating northern conditions and 32-33°C in the chambers simulating southern conditions. Daytime RH values were in the range of 93-96% (Table 2). Substantial variability in survival was evident among larvae of different genotypes both within and between regions (Figure 3). The ANOVA model successfully predicted arcsine transformed proportions of larvae surviving (***R***^***2***^ = 0.663, ***F*** = 26.87, df_1_ = 11, df_2_ = 150, ***p*** < 0.0001), with significant differences in larval survival among regions (***F*** = 68.31, df_1_ = 2, df_2_ = 150, ***p*** < 0.0001), and with greatest survival of WI_2_ larvae, but comparable survival of larvae from GA and MA (Figure 4). However, survival under northern vs. southern conditions showed a significant interaction with region of origin (***F*** = 5.16, df_1_ = 2, df_2_ = 150, ***p*** < 0.0068), so we analyzed the regions separately using factorial ANOVA (N/S x clutch). Larvae from different mothers in each region differed significantly in overall survival (GA, ***F*** = 23.78, ***p*** < 0.0001; WI, ***F*** = 8.15, ***p*** < 0.0009; MA, ***F*** = 24.57, ***p*** < 0.0001; df_1_ = 2, df_2_ = 48 in all comparisons). Larvae from GA and MA showed higher survival under northern than under southern conditions (GA, ***F*** = 17.18, df_1_ = 1, df_2_ = 48, ***p*** <0.0001; MA, ***F*** = 54.94, df_1_ = 1, df_2_ = 48, ***p*** < 0.0001). Survival under northern vs. southern conditions did not differ significantly for the larvae from WI (***F*** = 2.70, df_1_ = 1, df_2_ = 48, ***p*** = 0.1068), but the trend was in the same direction (Figure 4).

**Table 2:** **Daytime physical conditions in treatment chambers; experiment 2**

	Northern conditions			Southern conditions		
	Mean	SE	N	Mean	SE	N
GA larvae						
Temp (°C)	22.34	0.031	35	32.38	0.077	35
RH (%)	95.47	0.341	34	95.53	0.298	34
WI & MA larvae						
Temp (°C)	22.69	0.174	36	32.79	0.087	36
RH (%)	94.03	0.647	36	93.67	0.434	36

### Experiment 3

**Figure 5:**
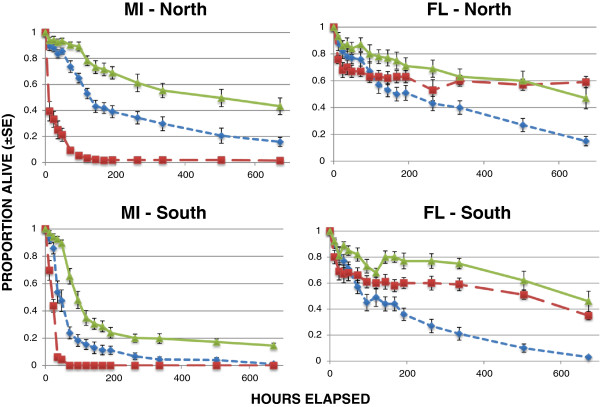
**Survival of** ***I. scapularis*** **larvae from three different mothers from each of two different regions under northern and southern conditions at moderate relative humidity.** Error bars are ± SE.

**Figure 6:**
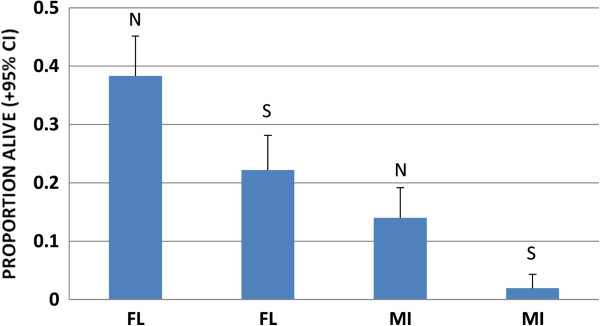
**Mean proportion alive after 28 days (+95% CI) of larvae from Florida (FL) and Michigan (MI) under northern (N) and southern (S) conditions.** Means backtransformed from arcsine transformed data.

Daytime temperatures were in the vicinity of 22 - 23°C in the chambers simulating northern conditions, and 32 - 34°C in the chambers simulating southern conditions. Relative humidities ranged from 81 - 86% under northern conditions and 81-82% under southern conditions (Table 3). Survival varied substantially both among larvae from different mothers within regions, and among larvae from different regions (Figure 5). The ANOVA model successfully predicted arcsine transformed proportional survival (***R***^***2***^ = 0.754, ***F*** = 43.87, df_1_ = 7, df_2_ = 100, ***p*** < 0.001). Survival differed among larvae from different mothers within regions (***F*** = 46.57, df_1_ = 4, df_2_ = 100, ***p*** < 0.0001), and overall between regions (***F*** = 83.18, df_1_ = 1, df_2_ = 100, ***p*** < 0.0001), with the FL larvae surviving longer than the MI larvae (Figure 6). Both FL and MI larvae survived longer under northern than under southern conditions (***F*** = 36.72, df_1_ = 1, df_2_ = 100, ***p*** < 0.0001). There was no interaction between effects of region of origin and north–south conditions (***F*** = 0.03, df_1_ = 1, df_2_ = 100, ***p*** = 0.339).

**Table 3:** **Daytime physical conditions in treatment chambers; experiment 3**

	Northern conditions			Southern conditions		
	Mean	SE	N	Mean	SE	N
FL larvae						
Temp (°C)	22.60	0.044	36	32.59	0.026	36
RH (%)	81.61	0.341	36	81.92	0.352	36
MI larvae						
Temp (°C)	22.36	0.046	36	33.13	0.082	35
RH (%)	85.83	0.355	36	81.067	0.454	34

## Discussion

Our results demonstrate clear differences in survival patterns among larval *Ixodes scapularis* from different regions of the northern and southern U.S. These regional differences are substantial, despite considerable variability of survival patterns among larvae from different mothers within regions (Figures 3, 5). Interestingly, survival patterns did not show clear north–south trends. In controlled trials, ticks from both northern and southern parts of the U.S. showed long survival from some sites and relatively short survival from others. Wisconsin larvae survived longer than larvae from South Carolina and Georgia, but also longer than larvae from Massachusetts (which showed similar survival to the Georgia larvae). Larvae from Florida survived longer than larvae from Michigan. Clearly, there is no consistent north–south gradient in these results.

The one consistent result from all trials was that larvae tended to survive longer under northern conditions than they did under southern conditions. This was true of all tick populations tested, whether from the north or from the south (Figures 1, 4, 6). This might be expected because the warmer southern temperatures would be expected to increase desiccation, putting physiological stress on the larval ticks, and because physiological processes tend to be more rapid at higher temperatures. Nymphs, which are considerably larger than larvae and have lower surface area to volume ratios, showed less in the way of regional effects than did larvae (Figures 1 and 2). Nevertheless, the shorter survival of larvae under southern conditions suggests that the southern environment is less congenial for survival of *I. scapularis* than the north. Indeed, some studies have suggested that *I. scapularis* population densities in the south are lower than in the north [22, 23]. Furthermore, this environmental difference could have imposed selective pressure on southern populations that has resulted in life cycle characteristics or behaviors in southern ticks that could influence pathogen transmission. For example, preliminary data suggest behavioral differences in southern compared to northern genotypes of *I. scapularis* that might influence host encounter and pathogen transmission (Kuczaj IM, Hickling GJ, Tsao JI, unpublished observations; presented at 13^th^ International Conference on Lyme Borreliosis and Other Tick-Borne Diseases, August 2013, Boston, MA). We hypothesize that the environmental differences between the north and the south have resulted in the evolution of adaptations in southern *I. scapularis* ticks that result in less transmission of Lyme spirochetes in southern environments. Further research will be needed to determine whether this hypothesis is correct, and what the relevant adaptations of southern *I. scapularis* populations might be.

The longer survival of Wisconsin compared to Massachusetts larvae in our study (Figure 4) suggests that there are ecologically important genetic differences between northeastern and north central populations of *I. scapularis*. These differences might be related to the necessity to survive harsher winter environments in the northern midwest than in the northeast. Gatewood *et al*. [24] described greater overlap of larval and nymphal phenologies in midwestern compared to northeastern populations, and related those differences to climate variables and to maintenance of different strains of *Borrelia burgdorferi* in the tick populations. These different bacterial strains differ in virulence, suggesting possible implications for human disease. Our results suggest that northeastern *I. scapularis* differ genetically from north central genetic types, and that these differences might contribute to the patterns described by Gatewood *et al*. [24]. Studies of survival and of other characteristics of ticks from additional northeastern and north central sites would be needed to determine whether these differences are, in fact, regional in nature.

Some authors have argued that the differences in Lyme disease incidence between northern and southern states result from differences in host communities, including greater host species diversity in the south [25], and the presence of lizards in southern but not in northern sites [2, 26]. Others have suggested differences in tick phenology, presumably resulting from the higher temperatures and longer growing season in the south [27], which might interfere with the efficient transmission cycle found in the north. Of course, these hypotheses are not mutually exclusive. Nevertheless, our results present an additional hypothesis. Perhaps southern conditions are generally less congenial than northern conditions for survival of *I. scapularis*, as was true for the larvae in our experiments. This could result in lower population densities in southern states [22, 28], which in turn would result in fewer ticks per host animal, and possibly other ecological and behavioral differences that might affect pathogen transmission. For example, the relatively poor southern conditions could affect phenologies by shortening activity periods, or they could select for host-seeking behaviors that would result in less transmission of pathogens among hosts of *I. scapularis,* and less attachment of this tick species to humans [29]. The overall warmer temperatures in the south might simply produce a marginal environment for this tick species, resulting in a less efficient enzootic cycle. This phenomenon might contribute, along with the many other environmental differences between the north and south, to the observed geographical patterns in Lyme disease incidence.

Recent modeling studies have suggested that warmer conditions due to climate change might result in expansion of the distribution of Lyme disease into more northern latitudes [30, 31]. Interestingly, our results suggest that these same environmental changes might result in less Lyme disease in the southern portions of its range. Clearly, additional studies of the behavioral, life history, and ecological differences between various genetic groups of *I. scapularis* are likely to provide insights into the geographical distribution of Lyme disease in North America.

## Conclusions

Survival patterns differed among larval *I. scapularis* ticks from different regions in eastern and central USA. These patterns were characterized by significant differences in length of survival among larvae from different mothers within regions, and overall differences among larvae from different regions. These overall differences did not follow a simple north–south pattern, despite the published north–south patterns in tick genetics. Larvae from both northern and southern regions and at various relative humidities survived longer under northern conditions of temperature and diurnal period than under southern conditions. Thus, southern conditions are less favorable for larval survival, which might favor selection for ecological or behavioral adaptations that lower the level of pathogen transmission by *I. scapularis* to humans.
